# Measuring Reference-Free Total Displacements of Piles and Columns Using Low-Cost, Battery-Powered, Efficient Wireless Intelligent Sensors (LEWIS2)

**DOI:** 10.3390/s19071549

**Published:** 2019-03-30

**Authors:** Marlon Aguero, Ali Ozdagli, Fernando Moreu

**Affiliations:** Department of Civil, Construction and Environmental Engineering, University of New Mexico, Albuquerque, NM 87131, USA; magueroinjante@unm.edu (M.A.); aozdagli@unm.edu (A.O.)

**Keywords:** low-cost, wireless, sensor, acceleration, structure, displacement, monitoring, railway bridge

## Abstract

Currently, over half of the U.S.’s railroad bridges are more than 100 years old. Railroad managers ensure that the proper Maintenance, Repair, and Replacement (MRR) of rail infrastructure is prioritized to safely adapt to the increasing traffic demand. By 2035, the demand for U.S. railroad transportation will increase by 88%, which indicates that considerable expenditure is necessary to upgrade rail infrastructure. Railroad bridge managers need to use their limited funds for bridge MRR to make informed decisions about safety. Consequently, they require economical and reliable methods to receive objective data about bridge displacements under service loads. Current methods of measuring displacements are often expensive. Wired sensors, such as Linear Variable Differential Transformers (LVDTs), require time-consuming installation and involve high labor and maintenance costs. Wireless sensors (WS) are easier to install and maintain but are in general technologically complex and costly. This paper summarizes the development and validation of LEWIS2, the second version of the real-time, low-cost, efficient wireless intelligent sensor (LEWIS) for measuring and autonomously storing reference-free total transverse displacements. The new features of LEWIS2 include portability, accuracy, cost-effectiveness, and readiness for field application. This research evaluates the effectiveness of LEWIS2 for measuring displacements through a series of laboratory experiments. The experiments demonstrate that LEWIS2 can accurately estimate reference-free total displacements, with a maximum error of only 11% in comparison with the LVDT, while it costs less than 5% of the average price of commercial wireless sensors.

## 1. Introduction

Today’s critical infrastructure is subjected to pressing demands, such as a long-life cycle, sustainability, and reliability. In addition, external factors, such as variability of service loads, unexpected overloading, and disaster scenarios, including unpredictable environmental conditions, can impede performance and cause civil engineering infrastructure to age at accelerated rates. Owners conduct maintenance operations following a standard schedule. Different rates of deterioration combined with standardized maintenance approaches sometimes result in unnecessary maintenance that adds to the life cycle costs, whereas inappropriate maintenance scheduling may lead to disasters and the loss of human lives. As a solution to costly scheduled maintenance, Structural Health Monitoring (SHM) systems have gained interest in the civil engineering community, since they can help inspectors to make informed decisions related to the prioritization of infrastructure maintenance using the health indexes of individual structures and selecting those with higher needs of intervention first [1]. In the last decade, with the rapid development of new technologies and innovations, which include advanced wireless networks, SHM applications have become more affordable and applicable [2,3,4]. With the recent advancement of SHM, engineers can equip infrastructure more appropriately to deal with pressing issues better by monitoring its responses and by being informed of its condition during and after extreme demands.

North American railroad managers are interested in informing their infrastructure management decisions to improve safety and to increase profits through maintenance prioritization. North American railroads carry approximately 40 tons of freight per person every year [5]. According to the estimates of the U.S. Department of Transportation, the demands for freight transported via the railway network will significantly increase by tonnage, up to 88% by 2035 [6]. The railroad has a dense network that connects 225,000 km (140,000 miles) of rail track with over 100,000 bridges; one railroad bridge per every 2.25 km (1.4 miles) of track [7,8]. Over half of North American railroad bridges are more than 100 years old and require regular maintenance [9]. The Railroad Bridge Working Group of the Railroad Safety Advisory Committee (RSAC) of the Federal Railroad Administration (FRA) estimated that 24% of the bridge length in the United States [10] is made of timber that is past its service life [11]. Based on this report, North American railroads decreased the percentage of timber bridges in half from 1993 to 2008 (from 36% to 24%). Based on conversations with Class I railroad bridge managers, in the coming decades there will still be a significant percentage of timber bridges that need to be replaced by steel and concrete to increase safety and productivity. Railroads apply weight restrictions and speed reductions on trains that cross aging railroad bridges to ensure operational safety, which limits the performance of operations and economic profit [6,12,13]. The modernization of the bridge network requires a financial investment equal to 3–5% of the total capital expenditures [14]. Railroad funds and resources are limited and decisions about maintenance, repair, and replacement (MRR) of the bridge network must be cost-efficient [15,16]. Based on conversations with Class I railroad bridge managers, 40% of the bridge-maintenance budget is directed to MRR of timber bridges. Railroad managers are interested in assessing and quantifying the conditions of timber bridge structures to increase the safety of their aging bridge network.

Railroad engineers need to obtain objective and valid information about the structural integrity of bridges in the field to improve the safety of railroad operations and to make informed, cost-effective MRR decisions. A major challenge for the reliability of the inspections performed by railroad engineers is the standard practice demanding annual visual inspection of bridges [7,8]. The American Railway Engineering and Maintenance-of-Way Association (AREMA) recommends collecting measurements during service operation for the evaluation of existing bridges [17]. AREMA recommends that vertical chord deflections of timber bridges under live loads be under L/250, where L is the span length (Chapter 7, Section 3.1.15 in Ref. [17]). Likewise, AREMA recommends limiting vertical deflections of steel bridges under L/640 (Chapter 15, Section 7.3.3 in Ref. [17]). Railroad managers are interested in collecting railroad bridge displacements during train traffic to inform the safety of train bridge crossing operations. The measurements of bridge responses under trains can be compared to thresholds or previous measurements to objectively make decisions about safety [16]. Bridge displacement is measured in general by means of Linear Variable Differential Transformers (LVDTs) or other contact-type sensors, but recording such responses remains a challenge because frequently a fixed reference frame is not available [18]. If noncontact displacements in the field were available to railroad managers, more objective data would be available to inform their MRR decisions.

In recent years, numerous researchers have developed reference-free displacements methods to collect displacements, but there is a lack of industry implementation. More specifically, engineers have developed noncontact SHM, such as Global Positioning Systems (GPSs) [19,20]; however, current affordable GPSs do not provide sufficiently accurate measurements for SHM purposes. Similarly, engineers have used Laser Doppler Vibrometer (LDV) [21] and vision-based SHM, based on both stationary cameras and Unmanned Aerial Systems (UASs) [22,23,24], but the applicability of these approaches is limited because LDVs are cost-prohibitive, whereas the vision-based SHM systems that operate on stationary cameras require appropriate locations for camera deployment, within the correct line-of-sight of civil structures, such as bridges. Furthermore, although UASs are not restricted by considerations related to the camera placement, they only provide displacements of structure that are relative to the camera motion [25,26]. Other researchers employ strain- or acceleration-based SHM methods to measure displacement indirectly. However, practical application by industry of such methods remains a technical challenge not affordable to owners and managers. For instance, Park et al. [27] observe that since the double integration of acceleration produces low-frequency drifts in the estimated displacements, high-pass filters must be applied to suppress the drifts. Moreover, as displacements become close to zero-mean processes, it may be difficult to apply strain for high-frequency modes. Recently, Ozdagli et al. [28] developed the low-cost, efficient wireless intelligent sensor (LEWIS) sensor, which provides reliable, accurate, and inexpensive measurements of displacement by utilizing only acceleration data. However, LEWIS performs poorly when the pseudo-static deflections are dominant in the response. Additionally, LEWIS lacked data storage capabilities and battery autonomy. Industry is interested to consider implementing sensors in their MRR decisions if sensors: (1) are low-cost; (2) can estimate total reference-free displacements; and (3) can be placed on the bridge with battery and storage capability.

This paper summarizes the development of a second generation of the low-cost, battery-powered, efficient wireless intelligent sensor (LEWIS2) that collects reference-free total displacements of timber railroad bridges. The acceleration data are used to obtain both the dynamic displacement and the pseudo-static rotation that is converted into pseudo-static displacement data. Researchers designed a test specimen consisting of a pile model representing an upside-down timber railroad bridge bent. The cap of the pile model was attached to a shake table. The footing of the pile model was attached to a rigid frame. In the experiment setup, the shake table excited the cap of the pile model with displacements of real railroad bridges under real traffic conditions. Researchers conducted five different train crossing events to validate the accuracy of LEWIS2 by estimating reference-free total displacement. After the six tests, the research team converted transverse accelerations and rotations captured with LEWIS2 into reference-free total displacements. The comparison between the transverse displacements estimated using LEWIS2 and the responses obtained with LVDT showed that LEWIS2 can measure reference-free total displacements with an error of less than 11%, which is viewed by railroad owners as acceptable for field displacement sensing. Consequently, the end user is interested in this level of accuracy to make decisions, which is currently not available in the field. Another innovation of LEWIS2 is that the sensor platform is powered by an affordable battery and can save the measured data onboard on a Secure Digital (SD) card providing data persistency for posterior analysis. In addition, LEWIS2 can be built with off-the-shelf components, which is affordable for any infrastructure owner, and it costs less than 5% of the average price of a commercial wireless sensor.

## 2. Low-Cost, Battery-Powered, Efficient Wireless Intelligent Sensor (LEWIS2)

This section introduces the low-cost, battery-powered, efficient wireless intelligent sensor LEWIS2 and describes its components.

### 2.1. Wireless Sensor Node Elements

Wireless SHM systems have important advantages over their conventional wired counterparts. First, wireless communication is highly economical, since a wireless sensor (WS) provides considerable cost reductions when compared with traditional and inflexible communication systems that require wiring and the labor associated with it [29]. Second, the cost efficiency of WS-based SHM translates into better quality of the assessment: it is possible to apply a dense array of wireless sensor nodes for the same cost, which in turn leads to a more accurate level of monitoring. Third, WS-based SHM systems implement real-time and autonomous monitoring [4,30,31]. Thus, advanced WS-based SHM systems enable reliable and efficient monitoring of bridges, buildings, bridges, dams, mines, wind turbines, oil rigs, pipelines, and other important structures [32,33].

**Figure 1:**
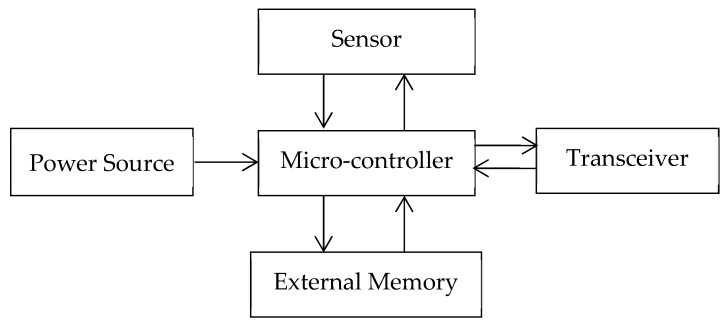
The interconnection of basic components of a wireless sensor.

Generally, a wireless sensor node is made of the following essential parts: a sensor, a microcontroller, an external memory, a transceiver, and a power source [34]. The sensor or sensing unit acquires data from the field and converts it into digital data. The microcontroller is used as a processor to acquire and process the sensor raw data to store the results to an external memory. The external memory may save a large amount of data, enabling posterior access by the user. The transceiver unit enables data sharing between the base station or other wireless nodes. The final element is a power unit, which consists of an energy source (capacitor, battery, or both). Depending on application requirements, further modules can be added. Figure 1 presents the way these elements are interconnected.

### 2.2. Elements of the Proposed LEWIS2

The previous section outlined the key components of wireless sensors. This section presents recommendations for specific components that could be provided for LEWIS2 on the basis of their characteristics and availability on the market.

### 2.3. Microcontroller

Arduino Uno R3 is an open-source microcontroller board, based on the ATmega328P microcontroller. The board contains the following components: 14 digital input/output pins (which are applied to read information from the sensor and to control the actuator, respectively), six of which can be applied as Pulse With Modulation (PWM) outputs, a USB connection, six analog inputs, a power jack, an In-Circuit Serial Programming (ICSP) header, a 16 MHz quartz crystal, and a reset button [35]. It can be powered from Alternating Current (AC) adapters, most USB chargers, or the USB port through a computer. Arduino Uno R3 has numerous advantages. It is flexible, features a user-friendly interface, and enables smooth communication between devices [36].

### 2.4. Sensor

LEWIS2 employs a low power consumption multichip module (MCM) MPU9250, produced by TDK InvenSense [37], headquartered in San Jose, CA, USA. The MPU9250 is a multichip module that comprises two dies that are integrated into one QFN (Quad-Flat No-leads) package. The sensor has a wide dynamic measurement range, and it is equipped with an accelerometer with a dynamically selectable measurement range that varies from ±2 g to ±16 g in all the three axes. The Arduino Uno uses the Inter-Integrated Circuit (I2C) interface to receive accesses to acceleration data sampled by the MPU9250. A breakout board produced by Adafruit Industries contains the sensor unit [35]. The board has been connected to the Arduino board through a breadboard with minimal soldering and wiring. To configure the settings of the sensor, the researchers use an open-source Arduino library developed by Adafruit Industries [38].

### 2.5. Transceiver

#### 2.5.1. Arduino Wireless SD Shield

Arduino Wireless SD Shield, produced by Arduino, is a device that performs two functions. First, it permits the Arduino board to perform wireless communication via a wireless module, in particular, the XBee transceiver module [35], but it can be also used with other modules that have the same footprint to form mesh networks. Second, it features a micro SD card socket, which permits its user to store digital sensor data. It also contains a preboard on the shield that can be used for prototyping circuits [35]. The micro SD socket included in the Arduino Wireless SD shield can be interfaced with the Arduino SD library. The two functions of the shield (storing and accessing data and interfacing with XBee transceiver modules) can be utilized independently and together.

#### 2.5.2. XBee Series 1 Module

XBee Series 1 Module is a very simple and reliable device that permits communication between computers, systems, and microcontrollers. It also supports point-to-point and multipoint networks and is convertible to a mesh network point. According to the Digi XBee documentation [39], the module has the range of 100 feet indoors and 300 feet outdoors within line-of-sight. It is very easy to set up and no prior configuration is required to operate it in peer-to-peer environments, which means that it can replace wired serial connections that were formerly used. The module is equipped with a wire antenna of excellent quality. The researchers selected the XBee module because it enables reliable wireless connection between LEWIS2 and other devices, such as a computer or a smartphone.

#### 2.5.3. XBee Explorer

XBee Explorer is a USB to serial base unit that can be applied in the Digi XBee line. The unit is very simple to use and is compatible with all the XBee modules, which include the standard and Pro versions of the Series 1 and Series 2.5.

XBee Explorer houses an FT231X USB-to-Serial converter, which translates the data between the XBee and the computer. It also contains a reset button, RSSI (signal-strength indicator), a voltage regulator to provide power to the XBee, a power indicator, and four light-emitting diodes (LEDs) that are helpful for debugging the XBee. To operate an XBee, it must be plugged to the XBee Explorer, and connected via a mini USB-cable to a computer or a cellphone. This gives direct access to the serial and programming pins located on the XBee unit [39].

### 2.6. Power Source

Nanotech Battery was used as the power source [40]. The most important specifications of the battery are listed in Table 1.

These properties make it a reliable battery that can be used to provide energy to the wireless sensor, so that it can work in an autonomous way. Importantly, the battery is rechargeable, so it can be charged with a solar panel.

### 2.7. External Memory

The SD card uses Arduino Wireless SD shield. The communication between the microcontroller and the SD card uses Serial Peripheral Interface (SPI). The researchers used the Arduino SdFat library, which supports FAT16 and FAT32 file systems on both standard and high capacity SD cards, and which secures read/write access to FAT16/FAT32 file systems on SD/ Secure Digital High Capacity (SDHC) flash cards. The researchers chose not to use the Arduino generic SD library because it is relatively slow.

**Figure 2:**
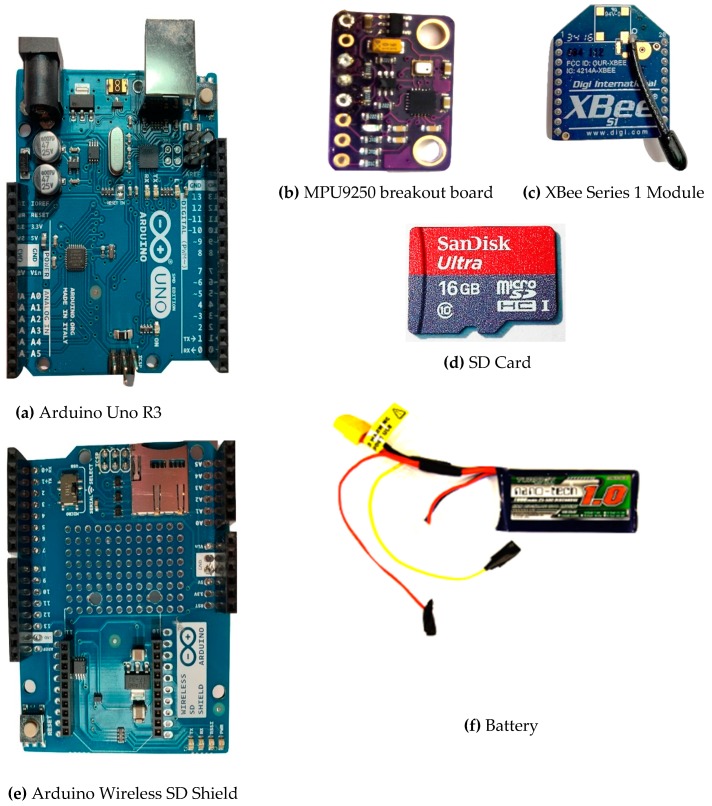
Components of the Arduino-based LEWIS2: (**a**) Arduino Uno R3, (**b**) MPU9250 breakout board, (**c**) XBee Series 1 Module, (**d**) Secure Digital (SD) Card, (**e**) Arduino Wireless SD Shield, (**f**) Battery.

**Figure 3:**
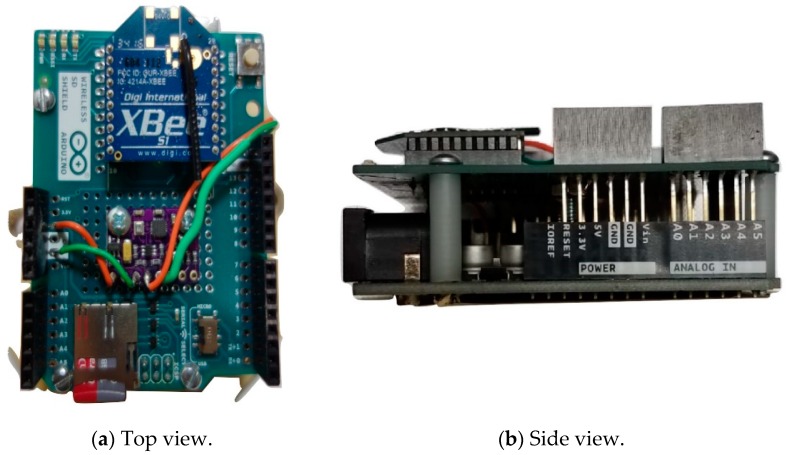
Assembled LEWIS2: (**a**) Top view, (**b**) Side view.

By means of summary, the elements of the proposed LEWIS2 are given in Figure 2 and the assembled LEWIS2 is illustrated in Figure 3. Additionally, Table 2 shows a cost summary of the elements of LEWIS2, with a description of each element.

## 3. Reference-Free Total Displacements

This section introduces the principles of estimating reference-free total displacements from an acceleration measurement. Such estimates are important because railroad bridges susceptible to asymmetric loadings may be subject to transverse displacements, which comprise dynamic and pseudo-static components. Since transverse displacements are directly related to the structural condition of bridges, they can be applied to determine bridge serviceability.

### 3.1. Principles of Total Displacement Estimation

The procedure for estimating total displacement is divided into the following three stages: (1) data collection, (2) data filtering, and (3) data fusion. The first stage is devoted to obtaining acceleration and inclination data via accelerometers. The second stage focuses on measurement filtering and it leads to the extraction of the pseudo-static and dynamic components. The dynamic component includes high-frequency responses triggered by rock-and-roll motions of the train, in which the centroid of the train may shift transversally and produce eccentric vertical loadings, which may lead to quasi-static transverse deflections of timber bridges [41]. The pseudo-static component is governed by such nonsymmetrical boundary conditions, triggered by the low-frequency displacement. In the third stage, extracted records are fused in order to obtain the total displacements.

Equation (1) will define the complete transverse displacements, $\Delta_{t}$:

(1) $$\Delta_{t}=\Delta_{d}+\Delta_{p}$$

where $\Delta_{d}$ and $\Delta_{p}$ represent the dynamic and pseudo-static components of the total transverse displacements, respectively. The subsequent sections describe how these components can be obtained.

### 3.2. Dynamic Displacement Estimation

To extract the dynamic displacement from an acceleration measurement, a finite impulse response (FIR) filter is used [42].

(2) $$\Delta_{d}={\left(L^{T}L+\lambda^{2}I\right)}^{-1}L^{T}L_{a}\bar{a}{\left(\Delta t\right)}^{2}=C\bar{a}{\left(\Delta t\right)}^{2}$$

(3) $$\lambda =46.81N^{-1.95}$$

where L is the diagonal weighting matrix. Moreu et al. [16], Ozdagli et al. [28,41], and Park et al. [43,44] provided a demonstration of the FIR filter in dynamic displacement estimation.

### 3.3. Principles of Inclination Sensing and Pseudo-Static Displacement Extraction

**Figure 4:**
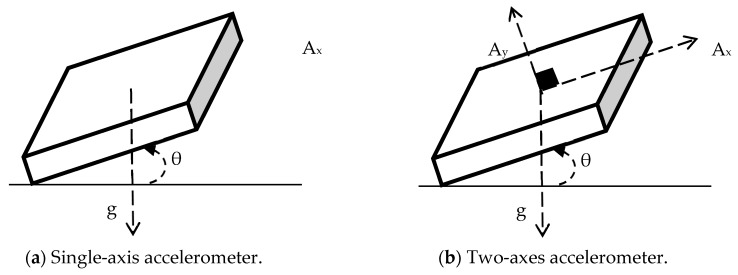
Sensing rotation using (**a**) a single-axis accelerometer, (**b**) a two-axes accelerometer.

Inclination sensing uses gravity and its projection on the axes of the accelerometers. This approach draws significant benefits from direct current (DC)-type accelerometers, which are capable of measuring static gravity vectors. For instance, the capacitive Micro-Electro-Mechanical System (MEMS)-type accelerometer is able to provide a 1-g response under gravitational acceleration. Figure 4 illustrates an accelerometer measuring the acceleration in its x-axis. When the accelerometer tilts by an angle, θ, the projection of the gravitational acceleration, g, on the x-axis of the sensor produces an output acceleration $A_{x}$, which is equal to the sine of the angle θ between the accelerometer x-axis and the horizon axis, which is orthogonal to the gravity vector. The resulting acceleration and angle can be formulated in the following way:

(4) $$A_{x}=g\times \sin\left(\theta \right)$$

(5) $$\theta =\sin^{-1}\left(\frac{A_{x}}{g}\right)$$

Single-axis inclination sensing may give imprecise readings when the resolution of the sensor is too low. The minimum required resolution is determined in the following way:

(6) R=g×(sin(N+P)−sin(N))

where *N* is the range of angle to measure, *P* is the minimum angle to measure [45], and R is the ratio between the maximal signal measured to the smallest part that can be resolved with an A/D (analog-to-digital) converter. When *N* is large and *P* is small, *R* turns to be small and causes a substantial error. In order to improve the accuracy of the inclination sensing, LEWIS2, in addition, measures the projection of the gravity vector onto the *y*-axis. In order to convert the measured acceleration in the *y*-axis $A_{y}$ to the inclination angle, the cosine of the angle θ between the *y*-axis and the gravity vector is calculated, as follows:

(7) $$A_{y}=g\times \cos\left(\theta \right)$$

(8) $$\theta =\cos^{-1}\left(\frac{A_{y}}{g}\right).$$

By combining Equations (4) and (7), it is possible to compute the ratio between $A_{x}$ and $A_{y}$ and establish the tangent of the inclination angle

(9) $$\frac{A_{x}}{A_{y}}=\frac{g\times \sin\left(\theta \right)}{g\times \cos\left(\theta \right)}=\tan\left(\theta \right).$$

Applying the inverse tangent function on both sides of Equation (8) gives the direct inclination.

(10) $$\theta =\tan^{-1}\left(\frac{A_{x}}{A_{y}}\right)$$

This approach is well-documented and used in many applications, including mobile phone orientation [45].

### 3.4. Pseudo-Static Displacement Estimation from Rotation Data

The FIR filter applied for computing zero-mean displacement cannot capture low-frequency pseudo-static characteristics of the displacements. In order to accurately measure the total displacement, the method developed in this paper reconstructs the pseudo-static component by calculating the inclination.

**Figure 5:**
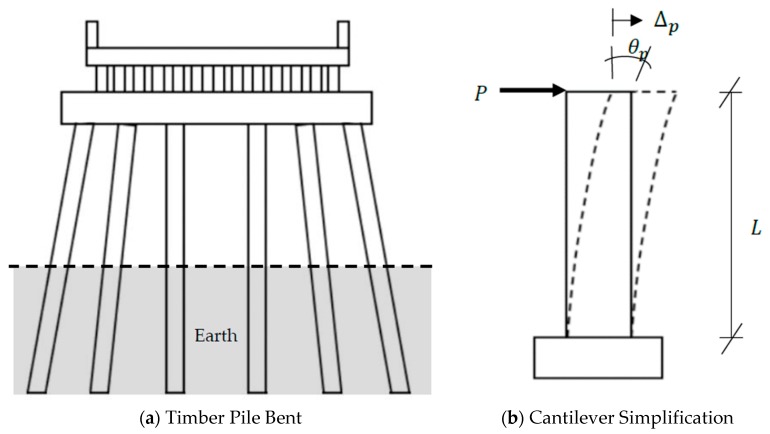
Pseudo-static displacement and pseudo-static rotation of a cantilever pile.

Deep pile foundations of the timber bents resist lateral loads because of soil resistance. Due to complex soil conditions, it is rather difficult to model the boundary conditions of embedded piles. However, in order to account for the soil resistance and enhance the reliability, the pile can be idealized as equivalent to a free-standing cantilever pile, which is commonly acknowledged in the railroad bridge industry as being representative of timber railroad bridge design and performance [46]. Figure 5 shows a simplified model of a timber railroad pile bent considering soil–structure interaction. Figure 5a shows the section view with six piles supporting the superstructure. Ozdagli et al. [41] provided a detailed analysis of cantilever simplification, which is shown in Figure 5b. Assuming a lateral load P that produces pseudo-static displacement $\Delta_{p}$, the resulting pseudo-static rotation will equal $\theta_{p}$.

The assumptions for this method ignore the fact that the railroad bridges in North America require a stiff deck that would restrain the free rotation of the end of the piles. However, the connection of pile caps to timber piles in railroad timber bridges is not rigid, consisting in general of long nails for shear in the pile/pile cap interfaces. Consequently, the rotation at the top of the pile is not restrained in general against small rotation. For the case of timber bridges in North America, this equation can be applied under this assumption.

In accordance with Euler–Bernoulli beam theory:

(11) $$\Delta_{p}=\frac{PL^{3}}{3EI}$$

(12) $$\theta_{p}=-\frac{PL^{2}}{2EI}$$

where L is the length of the cantilever pile; E is Young’s modulus; and I is the moment of inertia of the pile section. 

The combination of Equations (11) and (12) results in the ratio between $\Delta_{p}$ and $\theta_{p}$, which does not include material and section geometry properties, and only depends on $\theta_{p}$. The pseudo-static displacement yields to the following expression:

(13) $$\Delta_{p}=-\frac{2}{3}\theta_{p}L.$$

This paper provides a validation for the estimation of pseudo-static displacement observed because of asymmetric loading. Other potential contributions to pseudo-static displacement components, including shear, local effects, or other nonlinear behaviors, are not addressed in this study.

For typical bridge measurements, accelerations $A_{x}$ and $A_{y}$ contain both high-frequency and low-frequency responses. Thus, the inclination angle reproduced from these measurements may not converge to a pseudo-static value without preprocessing. In consequence, the estimated inclination still contains both dynamic and pseudo-static components. To attenuate the dynamic components and extract low-frequency components, a simple moving average (SMA) filter is applied. The resulting filtered data can be rendered in the following way

(14) $$\theta_{p}\left[i\right]=\frac{1}{n}\overset{n-1}{\underset{j=0}{\sum }}\theta_{t}\left[i+j\right]$$

where $\theta_{t}$ is total rotation containing both dynamic and pseudo-static components; i is the *i*th time step; and n is the number of points in the average, that is the size of the averaging window.

The size of the averaging window is adjusted on the basis of the frequency content of the total displacement. For instance, a train crossing a bridge at a faster speed may trigger higher frequency displacements [47]. In such scenarios, increasing n will be more effective in removing dynamic content from the input signal. As soon as the pseudo-static inclination is extracted, Equation (15) can be applied to calculate the pseudo-static displacements.

## 4. Experimental Validation

This section provides a description of the experimental setup that is used to validate the proposed method. The first part overviews the simplified cantilever beam model selected for the experiment. The subsequent part addresses the sensor placement and the instrumentation necessary for the method. Finally, this section provides an analysis and assessment of transverse displacements, accelerations, and rotations collected from the experiments to evaluate the performance of the developed method.

### 4.1. Experiment

**Figure 6:**
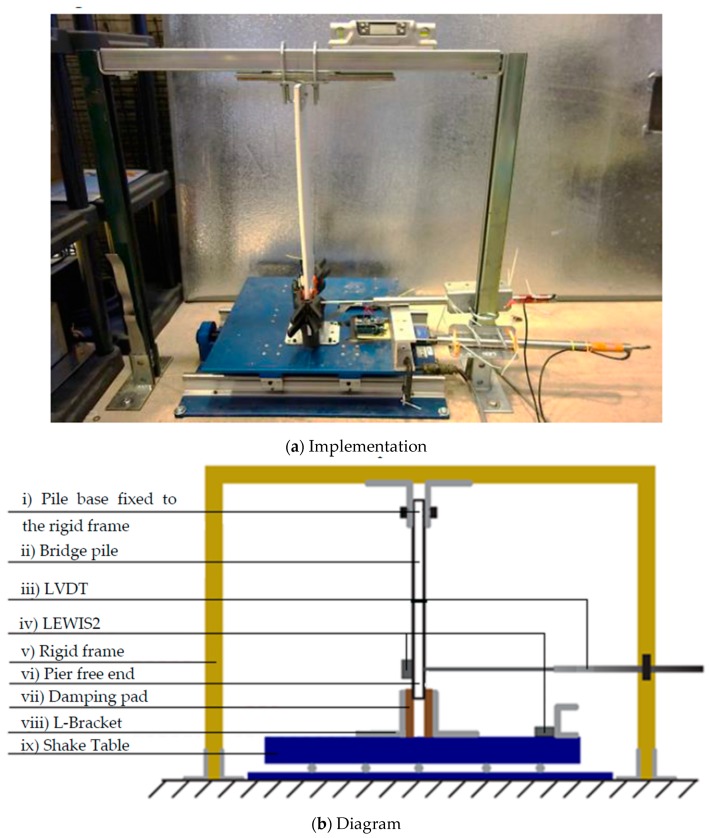
Instrumentation layout: (**a**) Implementation, (**b**) Diagram.

As shown in Figure 6, this study uses a cantilever pile that simulates the dynamic behavior of a timber railroad bridge pile (b.) The pile model is inverted in such a way so that the free end of the cantilever (vi) is excited by a shake table (ix) that is able to reproduce the bridge deck responses to train crossings. To move the pile tip freely, it is placed between two L-brackets (viii). The pile free end is restricted by sponge pads (vii) attached to L-brackets acting as equivalent rollers to damp out excessive vibration while preserving the cantilever’s behavior. Correspondingly, the fixed end of the pile (i) is secured to a rigid frame (v) that represents the stiff ground condition. The novelty of LEWIS2 resides in the proven portability and its low cost. The accuracy of a sensor of this cost that can collect total displacement wirelessly is also demonstrated in the next section of this paper. The validation of the sensor in simulated outdoor displacement tests provides evidence that LEWIS2 can accurately monitor reference-free total displacements of piles and columns, using the context of a railroad bridge train crossing as demonstration and validation.

### 4.2. Instrumentation

A Quanser Shake Table II, shown in Figure 6, with a maximum stroke of ±76 mm (±3 in) drives the pile. A low-cost, battery-powered efficient LEWIS2 (d) is placed on the pile. The accelerations measured by this sensor are applied to compute the pile rotation and extract the pseudo-static displacements, as explained in previous sections. The sensor measures both vertical and horizontal responses with respect to gravitational accelerations. The sensor selected for this experiment can deliver a true DC response and measure gravity and other types of sustained accelerations. Another LEWIS2 (d) is attached to a C-bracket on the shake table. A linear variable differential transducer LVDT (c) is placed to register the displacement of the pile at the point where the accelerometer pair is located.

The total displacement responses of the Bluford Bridge, a timber bridge located near Edgewood, Illinois, Chicago, USA, have been recorded by Moreu et al. [16]. These bridge displacement records were applied as the inputs to the shake table to excite the pile model. Table 3 provides a description of the five train velocities. The train speeds discussed herein are typical of and realistic for freight traffic in U.S. operations.

## 5. Results and Evaluation

**Figure 7:**
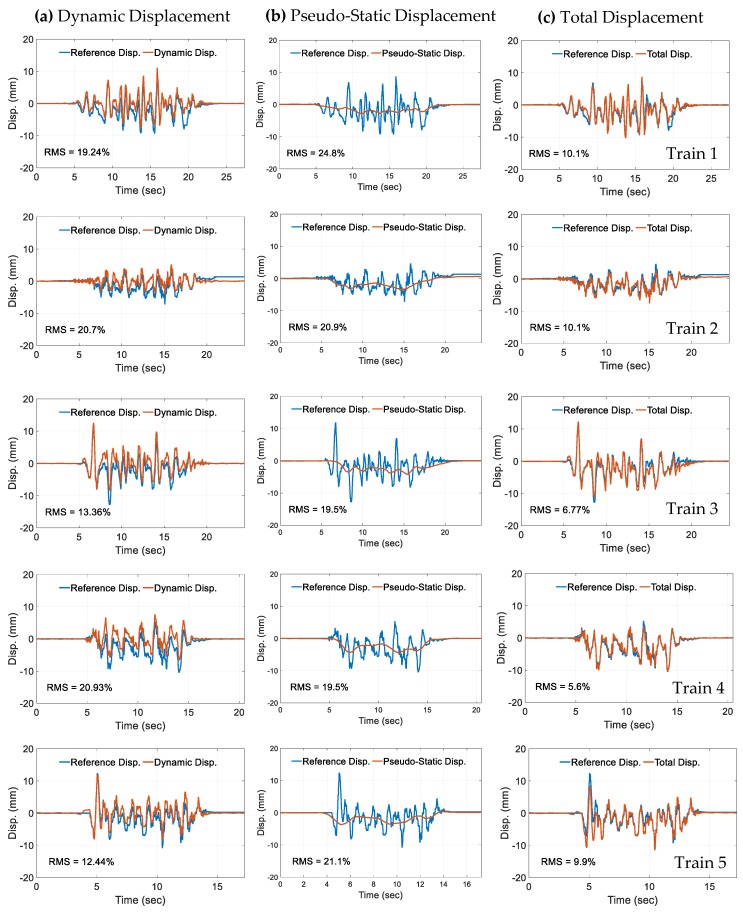
Reference and (**a**) Dynamic, (**b**) Pseudo-static, and (**c**) Total displacement: trains 1–5.

This section provides the outcomes of the experiment. The five bridge displacement profiles received on the field were used as input to the shake table. The shake table excited the structure and the commercial LVDT and the proposed LEWIS2 attached to it. The LEWIS2 sensor performs the following two actions: it first collects the acceleration in the three axes at the sampling rate of f_LS_ = 500 Hz, and, subsequently, it transmits the captured data to base stations using XBee. The researchers used the VibPilot to record the responses produced by the LVDT, at the sampling rate of f_VP_ = 1024 Hz. Next, for comparative purposes and verification, researchers resampled the data that had been collected by LEWIS2 at 1024 Hz and obtained the reference-free total displacement. In the last stage, the displacement data that had been derived from the LEWIS2 platform was compared with the reference signal collected with the LVDT. As shown in Figure 7, the dynamic displacement obtained with LEWIS2 only using the FIR filter is quite different than the reference displacement obtained with the LVDT. By contrast, the pseudo-static displacement showed in Figure 7 is very small in comparison with the dynamic displacement and the total displacement. By adding pseudo-static displacement to the dynamic displacement, researchers obtained similar displacements in both LEWIS2 and LVDT.

The performance indices are prepared in such a way so that smaller values indicate better performance, as shown in Equations (15) and (16).

(15) $$E_{1}=\frac{{\left|\Delta_{est}\right|}_{max}-{\left|\Delta_{meas}\right|}_{max}}{{\left|\Delta_{meas}\right|}_{max}}$$

(16) $$E_{2}=\frac{RMS\left(\Delta_{est}-\Delta_{meas}\right)}{RMS\left(\Delta_{meas}\right)}$$

The researchers obtained Root Mean Square (RMS) errors, which are presented in Table 4. To obtain the total displacement, we calculated the pseudo-static displacement with the procedure described in Section 3 and obtained the displacements shown in Figure 7. The sum of dynamic displacement and pseudo-static displacement gives us the total displacement shown in Figure 7. 

**Figure 8:**
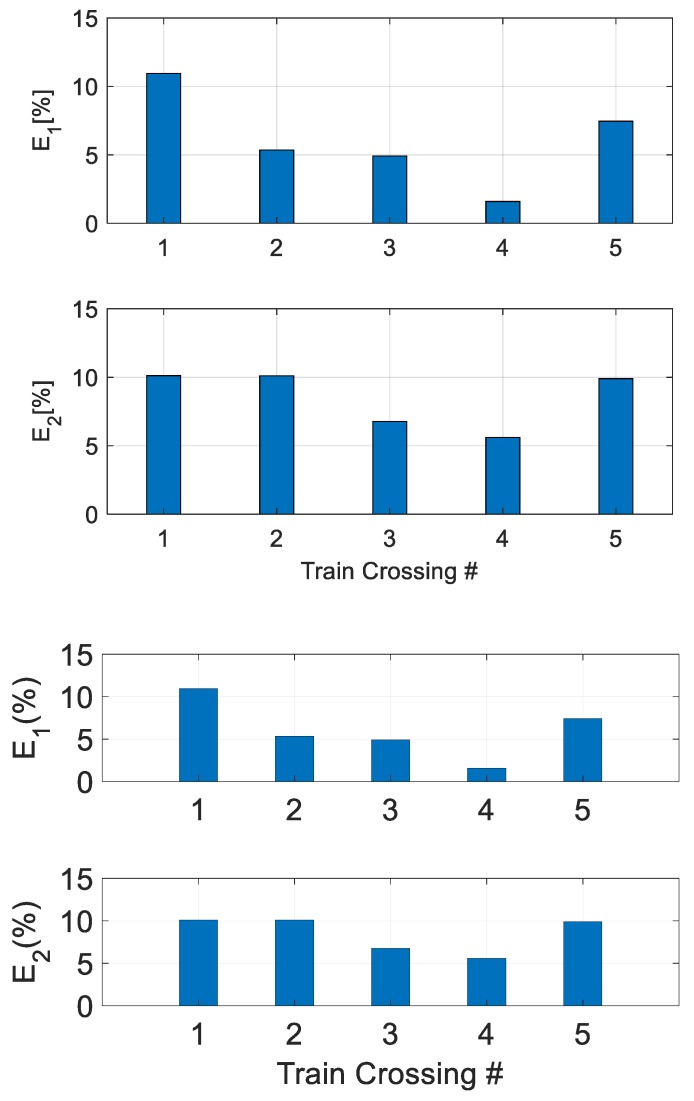
Performance evaluation results for total displacement estimation.

Researchers computed their performance indices in order to evaluate both the accuracy and the effectiveness of the LEWIS2 platform. Table 5 and Figure 8 show errors E1 and E2 in percentage terms for the five train crossing events. The results show that LEWIS2 estimates bridge displacements with an accuracy under 11%. The two highest values for E1 errors are under 11% and 8%, corresponding to the two largest displacement errors, for train numbers 1 and 5, respectively. The other three E1 errors are under 5.5%. The two highest values for the RMS (E2) error are under 11% for train numbers 1 and 2. The other three E2 errors range between 5% and 10%. In general, the railroad bridge managers accept 11% to be within an acceptable value of accuracy for field applications. Consequently, the results shown in this research are accurate for the specific application of railroad bridge management and field sensing of total displacements under train crossing events, which is currently not available in the field.

To summarize, the results of the experiment presented in this section demonstrate that the LEWIS2 is capable of providing accurate estimates of the total transverse displacement for railroad bridges. The LEWIS2 sensing platform does not require a fixed reference frame and is cost-effective: in comparison to commercial sensors, which cost approximately USD2000, all the components of LEWIS2 cost less than USD100 in total. Furthermore, LEWIS2 gives comparable performance results to those produced by the commercial LVDT.

## 6. Conclusions

This paper has addressed a new method to obtain total transverse bridge displacements of piles and columns by aggregating data obtained from multiple reference-free sensors and minimally relying on the structural properties. Researchers calculated the total displacements using the new LEWIS2 wireless sensing platform. LEWIS2 is an upgraded, battery-powered, data-storage-capable version of LEWIS, which can measure total displacements cost-effectively. The effectiveness of the new LEWIS2 system has been evaluated in a sequence of laboratory experiments. In the experimental setup, the researchers used a cantilever pile to simulate the dynamic behavior of a timber railroad bridge pile. The pile model was inverted so that the free end of the cantilever became excited by a shake table able to reproduce bridge deck responses to train crossings.

An FIR filter was used to extract dynamic displacement from acceleration. Likewise, an SMA filter was implemented to extract pseudo-static responses from the rotation data containing both slow and fast dynamic components. Later, the extracted data were converted to pseudo-static displacement using simple Euler–Bernoulli beam formulations. A comparison of the transverse displacements estimated using LEWIS2 with the responses received from LVDT indicates that LEWIS2 measures reference-free total displacements with an error less than 11%, which is deemed to be acceptable for field displacement sensing by railroad owners. The proposed new method, which utilizes the new LEWIS2 system, is particularly useful for structures, such as bridges, susceptible to asymmetric loading, which exhibit pseudo-static displacements. Significantly, the new method is cost-effective, as LEWIS2 can be built with off-the-shelf components, which in total cost less than 5% of the price of a typical commercial wireless sensor. The final conclusions of this research suggest developing and testing low-cost sensing approaches to collect displacements in the field, in order to increase the adoption by industry, such that objective information can be used to inform managers and owners of MRR decisions. The innovation of this research resides in the portability, cost-efficiency, and accuracy of an affordable sensor to measure reference-free total displacement for piles and columns under dynamic and pseudo-static loads.

## Figures and Tables

**Table 1:** Specifications of Nanotech 1.0 Battery [40].

Property	Value
Capacity	1000 mAh
Voltage	2S1P/2 Cell/7.4 V
Weight	60 g (including plug, wire, and case)
Dimensions	71 × 35 × 12 mm

**Table 2:** Essential sensing platform components of LEWIS2.

Element	Description	Price, $
Arduino Uno R3	Microcontroller	$4.00–25.00
MPU9250	Sensor	$9.99
XBee Series 1 Module	Wireless transmission module	$25.00
Arduino Wireless SD Shield	Communication XBee and Arduino Uno R3	$15.00
Battery	Nanotech LiPo 1000 mAh rechargeable battery	$6.33
SD Card	SanDisk Ultra 80 MBs MicroSD Card, 16GB	$10.00
	Total	$70.35–91.35

**Table 3:** Train crossing details.

Train Number	Velocity km/h (mph)
1	24.9 (15.5)
2	33.9 (21.0)
3	31.1 (19.3)
4	41.5 (25.8)
5	41.0 (25.5)

**Table 4:** Root Mean Square (RMS) Dynamic and Pseudo-Static Displacement.

Train Number	Dynamic Displacement		Pseudo-Static Displacement	
	$E_{1}\;\left(\%\right)$	$E_{2}\;\left(\%\right)$	$E_{1}\;\left(\%\right)$	$E_{2}\;\left(\%\right)$
1	19.61	19.24	68.70	24.83
2	28.70	20.70	50.98	20.86
3	2.83	13.36	67.23	19.50
4	27.62	20.93	57.80	19.48
5	4.77	12.44	70.26	21.12

**Table 5:** Performance results.

Train Number	$E_{1}\;\left(\%\right)$	$E_{2}\;\left(\%\right)$
1	10.94	10.11
2	5.35	10.10
3	4.91	6.77
4	1.60	5.60
5	7.45	9.90
